# Implicit Sensorimotor Adaptation Proceeds in Absence of Movement Execution

**DOI:** 10.1523/ENEURO.0508-22.2023

**Published:** 2023-08-02

**Authors:** Constance Pawlowsky, François Thénault, Pierre-Michel Bernier

**Affiliations:** Département de kinanthropologie, Faculté des Sciences de l’Activité Physique, Université de Sherbrooke, Sherbrooke, Québec, J1K 2R1, Canada

**Keywords:** motor commands, motor imagery, movement preparation, reaching, sensory prediction errors, visuomotor adaptation

## Abstract

In implicit sensorimotor adaptation, a mismatch between the predicted and actual sensory feedback results in a sensory prediction error (SPE). Sensory predictions have long been thought to be linked to descending motor commands, implying a necessary contribution of movement execution to adaptation. However, recent work has shown that mere motor imagery (MI) also engages predictive mechanisms, opening up the possibility that MI might be sufficient to drive implicit adaptation. In a within-subject design in humans (*n* = 30), implicit adaptation was assessed in a center-out reaching task, following a single exposure to a visuomotor rotation. It was hypothesized that performing MI of a reaching movement while being provided with an animation of rotated visual feedback (MI condition) would lead to postrotation biases (PRBs) similar to those observed when the movement is executed (Execution condition). Results revealed that both the MI and Execution conditions led to significant directional biases following rotated trials. Yet the magnitude of these biases was significantly larger in the Execution condition. To further probe the contribution of MI to adaptation, a Control condition was conducted in which participants were presented with the same rotated visual animation as in the MI condition, but in which they were prevented from performing MI. Surprisingly, significant biases were also observed in the Control condition, suggesting that MI per se may not have accounted for adaptation. Overall, these results suggest that implicit adaptation can be partially supported by processes other than those that strictly pertain to generating motor commands, although movement execution does potentiate it.

## Significance Statement

Directional biases were assessed following intermittent exposure to ±30° visuomotor rotations, when participants either executed a center-out reaching movement, merely imagined it, or performed a stereotyped inward movement to prevent motor imagery (MI). Interestingly, results revealed the presence of postrotation biases (PRBs) in all conditions, although they were largest in the execution condition. This work adds to the growing body of evidence suggesting that implicit adaptation may not strictly require overt execution but be supported to some degree by movement preparation alone.

## Introduction

Although several processes influence behavior when faced with a sensorimotor perturbation, implicit adaptation is thought to be primarily driven by sensory prediction errors (SPEs), the mismatch between predicted and actual sensory consequences of movements (Miall and Wolpert, 1996; Huang et al., 2011; Izawa and Shadmehr, 2011; Krakauer et al., 2019). These SPEs tend to be minimized on a trial-by-trial basis (Kasuga et al., 2013; Tan et al., 2014; Galea et al., 2015; Torrecillos et al., 2015; Savoie et al., 2018, 2020) by updating internal models of the body and the world, from which the sensory predictions arise (Miall and Wolpert, 1996). One hallmark of updated internal models is the presence of aftereffects on removal of a sensorimotor perturbation (Krakauer et al., 2019). Such automatic and nonstrategic biases in hand trajectory are also visible on the trial following an intermittent perturbation (Donchin et al., 2003; Diedrichsen et al., 2005; Galea et al., 2015; Torrecillos et al., 2015; Tan et al., 2016; Savoie et al., 2020; O.A. Kim et al., 2022), even when participants are instructed to ignore the visual error (Morehead et al., 2017; Maresch et al., 2021). These implicit postrotation biases (PRBs) stress the importance of updated predictions in sensorimotor adaptation.

Implicit adaptation has long been theorized to be linked to descending motor commands (Wolpert et al., 1998; Wolpert and Ghahramani, 2000; Krakauer et al., 2019), implying that movement execution is required for formulating predictions and generating SPEs. However, converging work suggests that adaptation may be more closely dependent on motor preparatory processes, rather than those that mediate execution per se. In support, Sheahan et al. (2016) showed that interference in learning two opposing force-field perturbations over the same movement can be prevented if each perturbation is associated with a different plan (i.e., a different follow-through movement), even if not executed. They suggested that adaptation hinges on neural states associated with movement planning, rather than those corresponding to execution (Sheahan et al., 2016). Interestingly, these authors also showed that this interference effect took place when the different follow-through movements were merely imagined (Sheahan et al., 2018), suggesting that motor imagery (MI) and motor preparation involve similar neural mechanisms.

In light of these results pointing to a key role of preparatory processes in adaptation, an intriguing open question is whether PRBs, a hallmark of SPE-dependent implicit adaptation (Savoie et al., 2020; O.A. Kim et al., 2022), can be elicited merely by MI. This possibility is especially interesting in light of recent work revealing that MI leads to sensory attenuation, a phenomenon thought to depend intimately on sensory predictions (Blakemore et al., 1998). Specifically, in a force-matching task Kilteni and colleagues (Kilteni et al., 2018) asked participants to press their right index finger on a sensor placed above their left index finger to match the perceived force received on the latter. In this active condition, participants perceived the reference force as less intense than in a passive condition, in which they relaxed their right index finger, suggesting that self-produced movements generate attenuation by accurately predicting their sensory effects. Critically, performing MI of the same action while receiving a force time-locked to the onset of MI led to near-identical sensory attenuation, indicating that MI generated sensory predictions of similar fidelity as real movements (Kilteni et al., 2018).

In this light, the present work tested the hypothesis that MI, if paired with an animation of perturbed visual feedback, is sufficient to induce sensorimotor adaptation. For that purpose, a visuomotor adaptation paradigm was used to assess PRBs following intermittent exposure to ±30° visuomotor rotations. To isolate the contribution of movement preparation to PRBs, three conditions were compared. First, in the Execution (EXE) condition, participants had to physically reach to the visual target following its presentation, while being provided with real-time visual feedback of the hand through a cursor. Second, in the MI condition, participants did not physically move but rather had to imagine themselves reaching the target following its presentation. Critically, the sensory feedback was emulated by the animation of the visual cursor, provided at each participant’s mean reaction time (RT). Finally, in the Control (CTL) condition, participants were also provided with the animated visual cursor following an individualized RT delay but had to execute a small stereotyped inward movement at target onset, regardless of its location. This was meant to prevent them from performing MI of the reaching movements. Given the aforementioned evidence, it was hypothesized that the EXE and MI conditions would generate direction-dependent PRBs, but not the CTL condition.

## Materials and Methods

### Participants

Thirty right-handed, healthy participants (15 women, mean age 25 ± 5), with normal or corrected-to-normal vision, took part in this within-subject study. All participants were naive to the experiment. All procedures were approved by the university’s ethics review board, before obtaining the written informed consent of participants. All participants came to the laboratory three times: first, to determine their MI ability, and then, to take part in two experimental sessions separated by 24 h (one for the MI condition, the other for the CTL condition).

### Experimental setup

**Figure 1.**
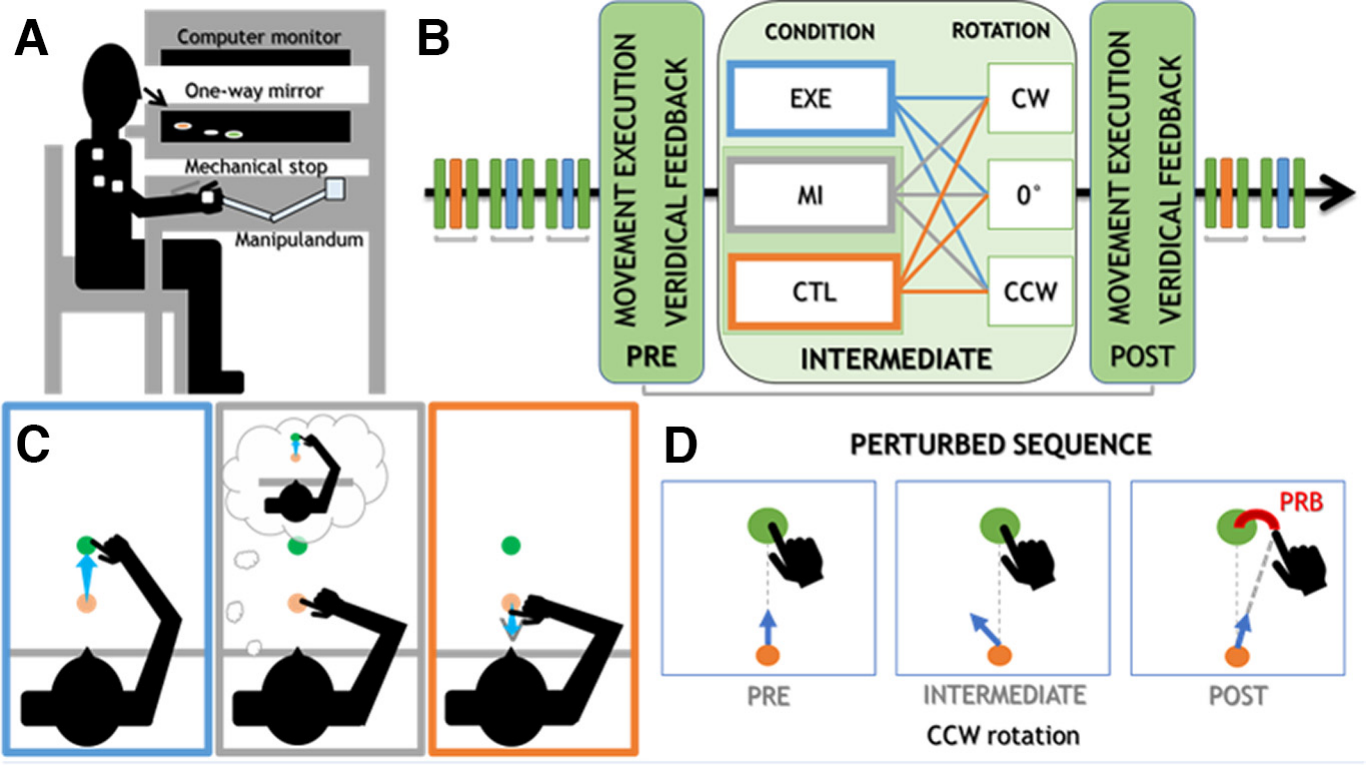
Overview of the methods. ***A***, Schematic representation of the experimental setup and electromyographic electrode placement (first dorsal interosseous, biceps brachii, the long head of triceps brachii, and anterior deltoid). ***B***, Trial timeline. The timeline of the experiment (black arrow) consisted of triplets of trials (colored sticks) in which the first and the third trials were compared to assess changes relative to the Intermediate trial, in which conditions and rotations were manipulated. Note that the MI and CTL trials were collected in two separate sessions, their order being counterbalanced between participants. ***C***, Experimental conditions. Participants made reaching movements toward the presented target (in blue: EXE condition), imagined reaching the target when it appeared without moving (in gray: MI condition), or made a small stereotyped movement toward themselves at target onset (in orange: CTL condition) to reach a V-shaped mechanical stop. ***D***, Schematic example of a perturbed triplet of trials showing a PRB following a rotation of the cursor (blue arrow) respective to hand displacement.

The experimental setup consisted of a table on top of which laid a custom-made manipulandum that was used by the participants to move a visual cursor and that allowed for the recording of the two-dimensional kinematics of hand movements at 1000 Hz (Fig. 1*A*). This manipulandum was made of two aluminum rods, two potentiometers, and a short handle at its mobile end. A 23-inch computer monitor (model VH238H, ASUS, resolution 1920 × 1080, refresh rate 75 Hz) was mounted face down 29 cm above a one-way mirror, itself positioned 29 cm above the table surface. Because the experiment was conducted in the dark, this device prevented participants from seeing their right arm while allowing them to view the visual stimuli on the same plane as their movement. Participants were sitting facing the table and resting their heads on a chin bar, to avoid head displacements. All procedures were run on MATLAB (v2017b, MathWorks), using functions from the psychophysics toolbox (Psychtoolbox) and custom codes.

### Experimental task

#### Reaching task overview

Participants were instructed to reach targets by moving a visual cursor with their right hand using the manipulandum. They were warned that the cursor would not follow their hand displacement on some trials – making their movement visually inaccurate. They were instructed to ignore it and aim so that their hand would cross the target (Morehead et al., 2017). As represented in Figure 1*B*, the full sequence of trials was divided into triplets (Pre, Intermediate, Post trials) toward one of three potential targets. The Intermediate trial of a triplet defined the condition [EXE, MI, or CTL (Fig. 1*C*); see below for specific instructions] and the presence of a rotation in the visual feedback [+30°, Counterclockwise (CCW); 0°, No Rotation; −30°, Clockwise (CW)]. The Pre and Post trials were both unperturbed and identical, which allowed us to assess PRBs by contrasting hand direction before (Pre) and after (Post) each Intermediate trial (Fig. 1*D*). No Rotation triplets were used as baseline values. On Pre and Post trials, participants made center-out movements to reach to the presented target by moving the manipulandum with their right hand. They were instructed to make a brief, ballistic and precise movement in the direction of the target. To make sure that participants moved through the target on each trial, they were asked to aim 2 cm beyond the presented target.

The choice of a PRB protocol was meant to minimize the contribution of other mechanisms known to mediate behavioral adaptation in typical “blocked” protocols in which performance improves over several trials and stabilizes at asymptote, including explicit re-aiming as well as model-free processes such as use-dependent plasticity and operant reinforcement (Huang et al., 2011).

#### Unfolding of trials

A trial was initiated when the visual cursor (white circle, ∅ = 0.85 cm) was placed on the starting base (orange circle, ∅ = 1 cm) for 700 ms. The starting base was positioned 26 cm in front of participants, aligned with their midline. Before target appearance, they were instructed to look at a fixation cross, located 6.5 cm in front of the starting base, along the midline (dimensions 0.3 × 0.3 cm). The initiation of each trial was accompanied by an auditory cue composed of four “beep” sounds (separated by 400 ms), the target being presented simultaneously with the last tone. These rhythmic auditory cues were used to help participants initiate their movements with consistent RTs on every trial, as well as inform them of the experimental condition on the upcoming trial. Four auditory tones were used, either with a low pitch for Pre, Post, and EXE trials (200 Hz, 80 dB, 40 ms), or a high pitch for MI and CTL trials (1000 Hz, 80 dB, 40 ms). Upon target presentation, participants were instructed to fixate it (green circle, ∅ = 2 cm) and to make the instructed action (see below for specific instructions). Although saccades were not monitored, performing a saccade to the appearing target is the natural behavior and all participants reported being able to do so fluently. The target could occupy one of three positions, presented in a pseudo-randomized order: left (15° CCW from midline), central (aligned with midline), or right (15° CW from midline). Regardless of position, the target was always presented 15 cm away from the starting base. Movement endpoint (ME) was defined as the moment when the visual cursor was displaced >15 cm from the starting base. On all trials, visual feedback was provided until movement endpoint (except in MI and CTL trials; see below, Practice block and animation of the visual feedback).

#### EXE trials

Although EXE trials were conceptually different from the Pre/Post trials, the same instructions and cues applied to these trials since they were indistinguishable from a participant’s standpoint. The objective was to move the hand in the target direction and to ignore the cursor if it did not follow the intended movement, without correcting it. These instructions were given to induce implicit visuomotor adaptation (Morehead et al., 2017). The intended movement was instructed as a straight, uncorrected, precise, and quick shooting movement (<400 ms), although participants were not required to execute their movement at maximal speed. On Pre/Post trials, visual feedback was veridical (i.e., the visual cursor mapped one-to-one with the hand movement). On EXE trials, visual feedback was either rotated (CW or CCW) or unperturbed (No Rotation) in pseudo-random order. The unperturbed trials allowed to set the 0° rotation as the baseline value of hand trajectory, as PRBs were not expected.

#### MI trials

On MI trials, participants were instructed to look at the presented target when it appeared, as in the EXE condition. However, instead of physically reaching it, they had to imagine moving their right hand to the target as if they were executing the reaching movement. They were specifically instructed to imagine the physical sensations of the movement they had to produce while keeping their eyes open and to focus on a first-person perspective. They were told not to visualize the action as if they were passively watching a movie scene. They were instructed to relax their arm but to imagine its displacement and the sensation of their hand moving on the table. They were warned not to contract their arm muscles nor to move, since the experimenter could monitor the surface electromyography (EMG) signal. These instructions were reiterated throughout the session. The critical manipulation was that on MI trials, an individualized animation of the cursor trajectory was provided so that participants had a visual input of the cursor reaching the target (unperturbed cursor) or being deviated (perturbed cursor; see below, Practice block and animation of the visual feedback).

#### CTL trials

In CTL trials, participants were asked to look at the target once it appeared, but instead of either reaching (EXE) or imagining reaching (MI) for it, they had to make a stereotyped inward movement with their right arm from the starting base to a V-shaped mechanical metal stop located 1 cm behind the starting base. The purpose of this condition was to prevent them from performing meaningful MI by “monopolizing” their motor system with a movement in a direction unrelated to the target being presented. Participants were explicitly instructed not to imagine reaching the target on these trials and to engage the biceps brachii (not only the wrist) even if the desired movement was of small amplitude. Critically, the same individualized cursor animations (perturbed or unperturbed) as in the MI condition were provided on these trials (see below, Practice block and animation of the visual feedback).

#### Practice block and Animation of the Visual Feedback

Participants familiarized themselves with the task during a 12-unperturbed-trial practice block, during which the three targets were presented four times in a pseudo-random order. This block was repeated until participants felt comfortable with the task. Then, exclusively in the first experimental session, participants completed a block of 36 unperturbed trials to create an animation of the cursor trajectory based on each participant’s kinematic profile. Thus, each target was presented in a sequence of four consecutive trials, with each sequence occurring three times per target. Trials were analyzed to reject those with RT > 1000 ms, movement times (MT) > 400 ms, and target errors (i.e., defined by movement endpoint superior to 4.5 cm relative to the center of the target). The animation was based on the remaining trials: for each target, the median values for RT and hand trajectory (thus MT) were computed. These animations (one per target) were provided either unperturbed (0° rotation) or perturbed (a ±30° rotation was implemented on the trajectory) in the MI and CTL conditions. The onset of the animation corresponded to the median RT of its constitutive trials.

Before each session, participants completed an unperturbed practice block made up of either 16 MI or 16 CTL trials (depending on the session), interspersed with 17 EXE trials, allowing participants to familiarize themselves with the session’s condition (MI or CTL) and associated auditory cues. Perturbed feedback was only provided during the experimental session.

#### Sequence of trials

A total of 1296 trials were spread over two experimental sessions, conducted on separate days. Unbeknownst to participants, the sequence of trials was split into 432 triplets: 16 triplets for each combination of rotations (3), conditions (3), and targets (3). Trial ordering was pseudo-randomized and was the same for all participants. It was also ensured that the sequence did not contain systematic ordering biases, in particular for the Rotation factor. The EXE triplets (whether perturbed or not) were equally spread over the two sessions, while the MI and CTL triplets were presented exclusively in one of the two experimental sessions. The motivation for separating the MI and CTL conditions in separate sessions was to reduce the cognitive load associated with the contingencies of the tasks. Indeed, we reasoned that both MI and the CTL movements would be better performed and interfere less with each other if they were conducted on separate days, rather than being intermixed. This was especially important since their purpose was orthogonal: the CTL condition was meant to prevent participants from performing MI. Thus, a mixture of EXE and MI triplets was provided on 1 d, and a mixture of EXE and CTL triplets was provided on the other day, with the order being counterbalanced across participants. In the end, 864 trials were Pre/Post trials (432 × 2, ∼67%), whereas 144 were EXE (∼11%), 144 were MI (∼11%), and 144 were CTL (∼11%) trials.

#### Motor imagery assessment

MI ability was assessed on participants’ first visit, before being exposed to the visuomotor task. A shortened version of the Motor Imagery Questionnaire-Revised Second Version (MIQ-RS; Gregg et al., 2010) was used, only including the kinesthetic items, and based on the French validated translation (Loison et al., 2013). Briefly, the MIQ-RS consists of performing a described movement once, then performing MI of the same movement without moving, and finally rating the ease of performing MI of the movement using a seven-point Likert scale. The larger the score, the easier the participants perceived the performance of MI. Participants were given the same instructions on how to perform MI during the questionnaire as during the visuomotor task (see above, Experimental task, MI trials). Following the familiarization block (see above, Practice block and animation of the visual feedback), an adaptation of the MIQ-RS was used to assess participants’ imagery ability within the visuomotor task (Reaching score): on a first trial, they were asked to reach a designated target (one of the three potential targets, verbally announced by the experimenter, before its appearance), then, on a second trial, to imagine reaching it. No visual feedback was provided on the second trial. Following the completion of the second trial, they rated their subjective MI ability with the MIQ-RS.

Following the completion of the experimental session in which MI was performed, three additional questions were answered by all participants regarding the quality of their MI, “I found it easy to imagine moving my arm during the experiment” (Ease score) and “I found that when I imagined moving my arm, the sensations seemed clear and vivid, almost like if the movement was real” (Vividness score) on a seven-point Likert scale, based on previously published work (Kilteni et al., 2018), and the frequency of MI (Frequency score; a score of 100 being equivalent to imagining moving on every MI trial).

#### Surface electromyography

To assess muscular activity and, in particular, to ensure that it was minimal when participants were asked to perform MI without moving, EMG was measured at four muscle locations [first dorsal interosseous (FDI), biceps brachii, the long head of triceps brachii and anterior deltoid] of the right arm. A reference electrode was placed on the C7 cervical vertebra. To improve the conductance of the signal, a soft abrasive gel (Nuprep, Weaver and Company) was first applied, before cleaning the skin with an alcohol pad. Marks were made on the participants’ skin in the first session to standardize the electrode placement across both sessions. EMG was recorded using Delsys’ Bagnoli-16 desktop system and Bagnoli DE-2.1 sensors (Delsys Inc.), and sampled at 1000 Hz. The EMG signal was monitored online during each experimental session to remind participants to remain relaxed and still on MI trials.

### Data processing

#### Trial rejection based on kinematics

Before analysis, we removed trials that were outliers based on RT, MT, and accuracy. For Pre, Post, and EXE trials, outliers based on RT and MT were detected per participant using the mean absolute deviation (±3 MAD; Leys et al., 2013). The conservative lower bound for RT was set to 100 ms to remove trials deemed too quick to allow for visual processing (Carlton, 1992; VanRullen and Thorpe, 2001), while the upper bound was set to 400 ms. The mean proportion of rejected trials based on RT and MT was 0.95 ± 3.88% across participants. Accuracy (cm) was determined as the absolute distance between the location of the hand at movement endpoint and the center of the aiming target. A trial was rejected when accuracy was beyond 4.5 cm. The mean proportion of rejected trials based on accuracy was 2.22 ± 3.26% across participants. As for the intermediate trials of the MI condition, a trial was rejected if a cursor displacement superior to 0.4 cm was recorded in the time interval between target appearance and the end of the animated feedback. This was infrequent, as the mean proportion of rejected MI trials was 2.49 ± 3.64% across participants (or on average 3.6 rejected trials out of the 144 possible MI trials). As for the intermediate trials of the CTL condition, a trial was rejected if an outward cursor displacement (i.e., toward the target instead of toward the body) was recorded. This was also infrequent, as the mean proportion of rejected CTL trials was 2.77 ± 4.08% across participants (or on average 4.0 rejected trials out of the 144 possible CTL trials). Whenever a trial met the rejection criteria, the triplet in which it was embedded was removed from the analysis. In total, among the 1296 trials, an average of 8.43 ± 3.95% of trials were rejected per participant (109 ± 51 trials).

To verify whether the proportion of rejected trials differed across our experimental factors, we submitted the mean proportion of rejected intermediate trials (EXE, MI, and CTL combined) to a three-way RM-ANOVA with factors Condition (EXE, MI, CTL), Rotation (Clockwise, No Rotation, Counterclockwise), and Target (Left, Central, Right). Results revealed no significant interactions or main effects (all *p* > 0.14), suggesting that rejected trials were distributed similarly across the different factor levels.

#### Kinematic data reduction

The main kinematic variable to measure reaching behavior was hand direction at peak tangential velocity (HDPV). It was defined as the angular difference (°) between the vector joining the starting base with the center of the target and the vector joining the starting base with the hand position at peak tangential velocity. Peak tangential velocity was chosen to reflect the movement planning process without online correction (Carlton, 1992; Tseng et al., 2007). In the present data, on average, the time to peak tangential velocity for Pre and Post trials was 125 ± 27 ms, corresponding to 75.71 ± 11.85% of MT. From here on, positive and negative HDPV angles respectively correspond to counterclockwise and clockwise directions relative to the target center. The average HDPV for Pre trials across all targets was 1.02 ± 1.15° (i.e., left of targets). To assess changes in HDPV, the Pre/Post change in hand direction of a given triplet was then calculated by subtracting the HDPV in the Post trial from that in the Pre trial. These Pre/Post changes were used to contrast corrective movements in response to the experienced rotations (i.e., the PRB). To test the robustness of the main results that used HDPV, additional analyses were also computed using the Pre/Post change in hand direction at movement endpoint.

To assess whether other movement kinematics could have been influenced by trial types, additional variables were computed. RT was defined as the time difference between target appearance and the moment when the cursor moved outside the starting base (i.e., movement initiation). MT was defined as the time between movement initiation and movement endpoint (i.e., the moment the 15-cm target distance was crossed). For Pre trials across all targets, the average RT was 305 ± 47 ms and the average MT was 172 ± 42 ms. The Pre/Post change for these variables was calculated as well.

#### EMG processing

To compare muscular activity across conditions and to confirm that participants did not produce significant muscular activity during MI trials, EMG activity was analyzed during the Intermediate trials. From the total recording for each session, EMG amplitudes were processed with MATLAB for each muscle, condition (EXE, MI, CTL), participant, and trial. A linear envelope detection was made first by removing the linear trends, computing the absolute value of the signal, then smoothing the rectified signal with a low-pass second-order Butterworth filter, with a 10-Hz cutoff. These smoothed, low-pass filtered values were then segmented into individual trials from 300 ms before target onset until movement endpoint. Each segment was baseline corrected by computing the ratio between the EMG linear envelope for each data point and the mean EMG linear envelope during baseline (e.g., before target onset). Hence a ratio of 1 means that muscular activity at any given time is no different from the mean baseline activity. To further prevent the influence of EMG artifacts on subsequent analyses, the integral of the linear envelope of each kept Intermediate trial was computed (from 300 ms before target onset until movement endpoint) and used to reject outliers across Conditions and Muscles using the MAD method (±3 MAD; Leys et al., 2013). Among the total 144 Intermediate trials per condition and following both kinematic and EMG rejections, there remained 86 ± 5%, 90 ± 4%, 89 ± 5%, and 90 ± 5% EXE trials; 86 ± 7%, 87 ± 7%, 85 ± 8%, and 88 ± 6% MI trials; as well as 85 ± 7%, 88 ± 6%, 85 ± 9%, and 87 ± 8% CTL trials, respectively for the FDI, biceps, deltoid, and triceps muscles. From these remaining trials, the median baseline-corrected ratio of Intermediate trials (from target onset to movement endpoint) was averaged across Conditions and Muscles and submitted to further analyses.

#### MI subjective ability

All scores obtained were averaged for each participant and transformed into a percentage. To assess whether Pre/Post changes in HDPV were predicted by MI ability, a general scale based on these scores was created (see Results).

### Statistical analyses

All analyses were performed on the statistical software Jamovi v.1.6.3. A sensitivity analysis revealed that 30 participants allowed detecting significant differences with an effect size (Cohen’s *d*_z_) of 0.7 at 90% power when using paired-samples two-tailed *t* tests with a Type I error α = 0.017. An α-value of 0.017 was used in anticipation that the significance threshold would decrease on correction for multiple comparisons (0.05/3 comparisons = ∼0.017). This was to ensure adequate power even when correcting for multiple comparisons.

For Pre/Post changes in HDPV, RT, and MT, RM-ANOVAs were conducted with factors Condition (EXE, MI, CTL), Rotation (Clockwise, No Rotation, Counterclockwise), and Target (Left, Central, Right). RM-ANOVA was chosen because of the multifactorial within-subject design used and its robustness to deviations from normality (Blanca et al., 2017). Violation of sphericity was assessed by Mauchly’s test and, when confirmed, *p*-values were corrected with Greenhouse–Geisser (for ε < 0.75, reported as *p*_GG_) or Huynh–Feldt corrections (for ε > 0.75, reported as *p*_HF_; Girden, 1992). Significant interactions were decomposed by analysis of simple effects. For η^2^_p_, values around 0.01, 0.06, and 0.14 are considered small, medium, and large, respectively (Lakens, 2013). When relevant, *post hoc* analyses using Bonferroni corrected paired *t* tests were performed (corrected *p*-values are reported as *p*_Bonferroni_). Cohen’s *d*_z_ values around 0.2, 0.5, and 0.8 are considered small, medium, and large, respectively (Lakens, 2013). The normality of the data were verified by the Shapiro–Wilk test (Mohd Razali and Yap, 2011). In case of the violation of normality, the Wilcoxon signed-rank test and its associated rank-biserial correlation (RBC) coefficient is reported, values around 0.10, 0.30, and 0.50 indicate small, moderate, and large association, respectively (Cohen, 1988).

For MI scores, the reliability between the five subjective scores was assessed using McDonald’s ω (Hayes and Coutts, 2020), to build a general MI ability score. Bivariate correlation analysis was used to evaluate the relationship between the MI scores and the Index of Pre/Post change in HDPV in the MI condition.

Muscular activity was compared between Conditions by performing pairwise comparisons on the median ratio of EMG activity relative to baseline, with Bonferroni correction for multiple comparisons following the same procedure as described above. In addition, EMG activity was compared with 1 on MI trials to confirm that it was not different compared with baseline (i.e., when no muscle activity was produced).

The α threshold for all statistical tests was set at 0.05 (corrected *p*-values are reported whereby necessary). Data are reported as mean (M) ± standard deviation (SD).

## Results

### Potential session effect in the EXE condition

Because EXE trials were spread over 2 consecutive days, we first assessed whether participants’ behavior was similar in both sessions. To this end, RM-ANOVAs with factors Session, Target, and Rotation were performed on HDPV, RT, and MT, only including the EXE trials. It was expected that there would be no main or interaction effect including the Session factor. After verifying sphericity, results revealed neither an effect of Session (all *p*-values > 0.444) nor an interaction including the Session factor (all *p*-values > 0.107) for HDPV, RT, and MT. These results are summarized in Table 1. In absence of a Session effect, the data of both sessions were pooled together in subsequent analyses.

**Table 1:** Effects of session, target, and rotation on the kinematic variables of the EXE trials (*n *= 30)

		PV	RT	MT
Session (2) × Target (3) × Rotation (3)	*F*_(4,116)_	0.35	0.69	0.83
	*p*	0.785 (GG)	0.597	0.468 (GG)
	η^2^_p_	0.01	0.02	0.03
Target × Rotation	*F*_(4,116)_	1.65	**46.35**	**29.5**
	*p*	0.185 (GG)	**<0.001**	**<0.001**
	η^2^_p_	0.05	**0.62**	**0.5**
Session × Rotation	*F*_(2,58)_	1.03	0.6	0.5
	*p*	0.363	0.554	0.552 (GG)
	η^2^_p_	0.03	0.02	0.02
Session × Target	*F*_(2,58)_	2.41	0.73	0.9
	*p*	0.107 (HF)	0.488	0.378 (GG)
	η^2^_p_	0.08	0.02	0.03
Rotation	*F*_(2,58)_	2.39	**60.08**	**37.46**
	*p*	0.129 (GG)	**<0.001 (GG)**	**<0.001 (HF)**
	η^2^_p_	0.08	**0.67**	**0.56**
Target	*F*_(2,58)_	2.65	**122.37**	**82.15**
	*p*	0.081 (GG)	**<0.001 (HF)**	**<0.001 (HF)**
	η^2^_p_	0.08	**0.81**	**0.74**
Session	*F*_(1,29)_	0.19	0.6	0.05
	*p*	0.667	0.444	0.817
	η^2^_p_	0.01	0.02	0

When the sphericity assumption is not met, *p*-values are corrected following Greenhouse–Geisser (GG) or Huynh–Feldt (HF) procedures. Bold values denote statistically significant effects.

### Pre/Post change in hand direction at peak tangential velocity

**Figure 2.**
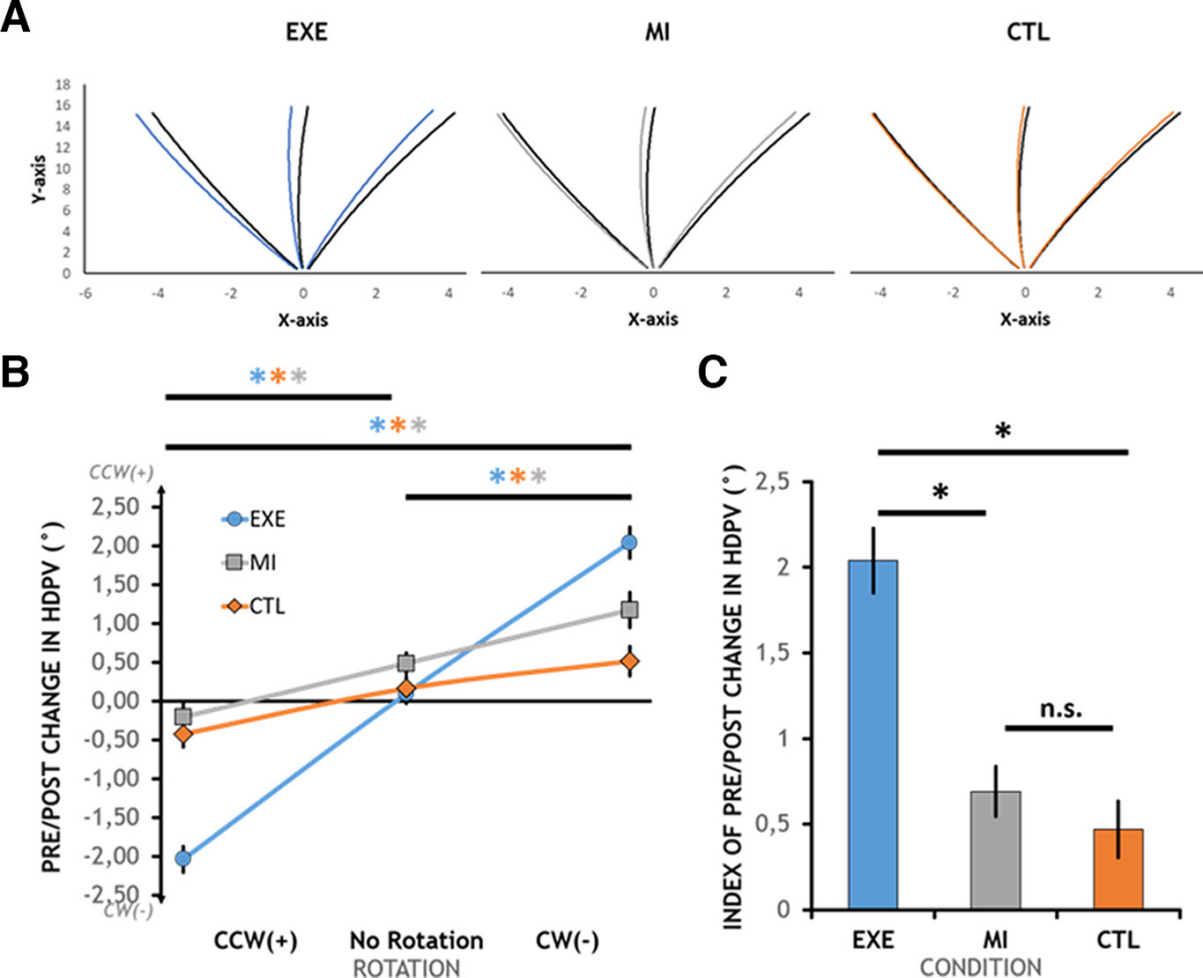
Pre/Post change in HDPV. ***A***, Mean trajectories of the Pre (black lines) and Post (colored lines) trials for each condition and each target. For clarity, only the clockwise (CW) rotation is shown. Hand trajectories are shifted CCW in all conditions, reflecting the presence of PRBs. ***B***, Interaction effect of Rotation × Condition on the mean Pre/Post change in HDPV (°). Mean differences are reported relative to Condition and Rotation of the Intermediate trials. Negative directions are clockwise (CW). ***C***, Effect of Condition on the Index of Pre/Post change in HDPV (°). Mean differences are reported relative to the Condition of the Intermediate trial and computed based on the Pre/Post change in HDPV following No Rotation-baseline correction. Error bars correspond to a 95% confidence interval. Significant differences are reported (*). N.s., nonsignificant difference.

The main goal of this work was to test whether sensorimotor adaptation occurs in absence of movement execution, as assessed by the presence of PRBs. The mean trajectories of the Pre and Post trials are presented in Figure 2*A*. The black trajectories correspond to the Pre trials and the colored trajectories correspond to the Post trials, for each condition and target. For clarity, only the clockwise (CW) rotation is shown. As can be seen, the EXE condition is associated with potent PRBs as depicted by a shift in hand trajectories opposite to the direction of the rotation (i.e., CCW in this case). Most importantly, both the MI and CTL conditions are associated with smaller, yet clearly discernable PRBs.

Regarding Pre/Post changes in HDPV, the results revealed no significant three-way interaction of Target × Condition × Rotation (*F*_(8,232)_ = 1.78, *p*_GG_ = 0.113, η^2^_p_ = 0.06), but a significant Condition × Rotation interaction with a large effect size (*F*_(4,116)_ = 74.31, *p* < 0.001, η^2^_p_ = 0.72), a significant Target × Condition interaction (*F*_(4,116)_ = 4.85, *p* = 0.001, η^2^_p_ = 0.14), as well as significant main effects of Rotation (*F*_(2,58)_ = 282.27, *p*_GG_ < 0.001, η^2^_p_ = 0.91) and Condition (*F*_(2,58)_ = 11.44, *p* < 0.001, η^2^_p_ = 0.28).

The results for each condition and rotation are presented in Figure 2*B*, with data collapsed across targets as justified by the lack of three-way interaction. Breakdown of the Condition × Rotation interaction of primary interest revealed simple effects of Rotation in all three Conditions (EXE: *F*_(2,58)_ = 322.3, *p*_HF_ < 0.001, η^2^_p_ = 0.92; MI: *F*_(2,58)_ = 55.74, *p* < 0.001, η^2^_p_ = 0.66; CTL: *F*_(2,58)_ = 22.37, *p* < 0.001, η^2^_p_ = 0.43), with large effect sizes, being the largest for the EXE condition.

Pairwise comparisons, revealed that, in the EXE condition, the mean difference between CCW-rotated (M = −2.04 ± 0.74°) and unperturbed triplets (0.09 ± 0.46°) was significant (*t*_(29)_ = 17.52, *p*_Bonferroni_ < 0.001, *d*_z_ = 3.20), indicating that participants displayed CW PRBs following CCW rotations. Similarly, the Pre/Post change in unperturbed triplets was significantly different from CW-rotated triplets (2.04 ± 0.75°), indicating CCW PRBs following CW rotations (*t*_(29)_ = 12.31, *p*_Bonferroni_ < 0.001, *d*_z_ = 2.25). Not surprisingly, PRBs following CW and CCW triplets were also significantly different from each other (*t*_(29)_ = 21.02, *p*_Bonferroni_ < 0.001, *d*_z_ = 3.84).

Qualitatively similar results were observed for MI albeit with decreased magnitude: unperturbed triplets (0.49 ± 0.39°) were significantly different from CCW-rotated triplets (−0.21 ± 0.60°; *t*_(29)_ = 5.97, *p*_Bonferroni_ < 0.001, *d*_z_ = 1.09), and from CW-rotated triplets (1.17 ± 0.64°; *t*_(29)_ = 5.44, *p*_Bonferroni_ < 0.001, *d*_z_ = 0.99). PRBs following CW-rotated and CCW-rotated triplets were also significantly different from each other (*t*_(29)_ = 9.34, *p*_Bonferroni_ < 0.001, *d*_z_ = 1.70).

In the CTL condition, a pattern similar to that observed in MI trials was observed. Specifically, unperturbed triplets (0.18 ± 0.55°) were significantly different from CCW-rotated triplets (−0.42 ± 0.63°; *t*_(29)_ = 4.67, *p*_Bonferroni_ < 0.001, *d*_z_ = 0.85), and from CW-rotated triplets (0.51 ± 0.70°; *t*_(29)_ = 2.63, *p*_Bonferroni_ = 0.041, *d*_z_ = 0.48). PRBs following CW and CCW-rotated triplets were also significantly different from each other (*W*_(29)_ = 22.00, *p*_Bonferroni_ < 0.001, RBC = 0.91).

Overall, these results show that significant PRBs occurred in all conditions, with directions opposite to the encountered rotation.

To test the robustness of this finding, PRBs were also computed using hand direction at movement endpoint (ME) instead of at peak velocity (at 172 ms instead of 125 ms). Results revealed a three-way interaction (*F*_(8,232)_ = 2.74, *p*_GG_ = 0.019, η^2^_p_ = 0.09), as well as a Condition × Rotation interaction (*F*_(4,116)_ = 84.74, *p*_HF_ < 0.001, η^2^_p_ = 0.75) and a Target × Condition interaction (*F*_(4,116)_ = 6.22, *p* < 0.001, η^2^_p_ = 0.18). There were also main effects of Rotation (*F*_(2,58)_ = 256.56, *p*_HF_ < 0.001, η^2^_p_ = 0.90), Condition (*F*_(2,58)_ = 6.06, *p* = 0.004, η^2^_p_ = 0.17), and Target (*F*_(2,58)_ = 12.04, *p*_GG_ < 0.001, η^2^_p_ = 0.29). Breakdown of the interaction into simple main effects of rotation for each condition confirmed the pattern of findings observed for HDPV, with significant PRBs being observed in all conditions, in directions opposite to the rotation.

As for the Target × Condition interaction observed in both analyses, it was attributable to the fact that mean PRBs (collapsed across Rotations) were consistently near zero across targets for EXE, whereas they were shifted CCW for the right target in the MI and CTL condition. Given that the next analysis (Index of Pre/Post change in hand direction at peak tangential velocity) was meant to control for this condition-dependent offset, this interaction will not be broken down further here.

### Index of Pre/Post change in hand direction at peak tangential velocity: absolute value relative to No Rotation-baseline

The net magnitude of the PRBs across conditions was then compared. However, as can be seen in Figure 2*B*, the Pre/Post change in HDPV in unperturbed triplets was shifted CCW in the MI (0.49 ± 0.39°), EXE (0.09 ± 0.05°), and CTL (0.18 ± 0.17°) conditions. Paired-comparisons using either Wilcoxon or *t* tests, depending on the normality of the data, and Bonferroni correction for multiple comparisons, revealed that these shifts were significantly different between MI and EXE (*t*_(29)_ = 3.65, *p*_Bonferroni_ = 0.003, *d*_z_ = 0.67), but not between MI and CTL (*t*_(29)_ = 2.31, *p*_Bonferroni_ = 0.084, *d*_z_ = 0.42), nor EXE and CTL (*W*_(29)_ = 191.00, *p*_Bonferroni_ = 1, RBC = −0.18).

Hence, given these differences and the fact that unperturbed triplets were considered as a reference, the Pre/Post change in HDPV in unperturbed triplets was subtracted from the Pre/Post change in HDPV in each rotation. In addition, the sign of the CCW shift was inverted in all three conditions, and submitted to a two-way RM-ANOVA with factors Condition and Shift. The analysis of the resultant Index of Pre/Post change in HDPV revealed no Condition × Shift interaction (*F*_(2,58)_ = 0.33, *p* = 0.722, η^2^_p_ = 0.01), no main effect of Shift (*F*_(1,29)_ = 2.09, *p* = 0.159, η^2^_p_ = 0.01), but a significant main effect of Condition (*F*_(2,58)_ = 118.30, *p* < 0.001, η^2^_p_ = 0.49). Figure 2*C* shows that, when collapsed across the Shift factor, the Index of Pre/Post change in HDPV was largest for the EXE condition (2.04 ± 0.53°), followed by the MI condition (0.69 ± 0.41°), and the CTL condition (0.47 ± 0.46°). Pairwise comparisons revealed significant differences between EXE and MI (*t*_(29)_ = 13.55, *p*_Bonferroni_ < 0.001, *d*_z_ = 2.47), EXE and CTL (*t*_(29)_ = 14.02, *p*_Bonferroni_ < 0.001, *d*_z_ = 2.56), but no significant difference between MI and CTL (*t*_(29)_ = 1.86, *p*_Bonferroni_ = 0.073, *d*_z_ = 0.34).

Overall, these analyses show that PRBs were larger in the EXE condition compared with the MI and CTL conditions, which did not differ from each other.

To gain insight into whether the PRBs observed in each condition are subserved by similar mechanisms, we then looked at between-participant correlations in the magnitude of PRBs across conditions. Results revealed a trend for a significant positive correlation between EXE and MI (*r* = 0.38, *p* = 0.06), but not between EXE and CTL (ρ = 0.15, *p* = 0.42) or MI and CTL (ρ = −0.17, *p* = 0.36). This can be tentatively interpreted as more similar underlying mechanisms supporting PRBs between EXE and MI, while CTL would be more dissimilar.

### Comparison of the duration of visual feedback in the EXE trials and the MI and CTL trials

Because the animation of the visual feedback was derived from participants’ own reaching movements (see methods), the spatial features of the visual feedback were very similar across conditions. However, despite careful precautions, there may have been subtle differences in the timing at which it was presented across conditions. To verify this, mean RT and MT values of the EXE trials and the trials composing the animation were computed for each participant. Mean RT and MT values of the EXE trials were, respectively, 226 (±31) and 207 (±31) ms, while the trials composing the animation were, respectively, 288 (±47) and 166 (±36) ms. Pairwise comparisons revealed that these differences (ΔRT: 62 ms; ΔMT: 41 ms) were significant (RT: *W*_(29)_ = 3.00, *p* < 0.001, RBC = 1; MT: *t*_(29)_ = 13.68, *p* < 0.001, *d*_z_ = 2.50).

Hence, in the EXE condition, participants were provided with visual feedback earlier and for a longer duration than in the MI or CTL conditions. This is a potential limitation, as it may have accounted for some of the differences in PRB magnitude between trials with real-time feedback (that is, EXE trials) versus those with animated feedback (that is, MI and CTL trials). In an attempt to evaluate this potential influence, we performed a bivariate correlation analysis across the 30 participants in which we compared the difference in RT between EXE and that of the animations (i.e., used in the MI and CTL conditions) and the difference in PRB between the EXE and the MI and CTL conditions (pooled together). This analysis was also done for the MT data. Results revealed that none of the correlations were significant (RT: *F*_(1,28)_ = 0.90, *p* = 0.352, *r* = 0.03; MT: *F*_(1,28)_ = 0.67, *p* = 0.421, *r* = 0.02). This indicates that differences in the timing of the visual feedback across conditions were essentially unrelated to the differences in PRB observed across conditions, thus mitigating this potential limitation.

### Subjective ability to perform motor imagery and magnitude of postrotation bias

Since MI ability has been linked with neural and motor improvements following MI training (Avanzino et al., 2015; Williams et al., 2012), it was hypothesized that MI ability might have been related to the degree of adaptation across participants. A unique MI ability scale was built to test whether it predicts the magnitude of the Index of MI Pre/Post change in HDPV. Even if individual scores of subjective MI ability showed variability (minimum-maximum values: MIQ-RS: 33–100%, Reaching: 40–100%, Ease: 29–100%, Vividness: 14–100%, Frequency: 50–100%), all scores were on average superior to 50% (for MIQ-RS: 69 ± 18%, Reaching: 73 ± 16%, Ease: 70 ± 20%, Vividness: 61 ± 23%, Frequency: 78 ± 15%). Significant correlations were found between scores, as assessed by Pearson’s r or Spearman’s ρ when the assumption of normality was not met (see Table 2). Thus, a unique scale was built, based on the average of these five items for each participant. This general MI ability score was normally distributed (Shapiro–Wilk test 0.97, *p* = 0.436). Its reliability was assessed by McDonald’s ω, giving a general value of 0.78, considered as being acceptable (Hayes and Coutts, 2020). The mean score was 70.24 ± 13.46%, suggesting that participants considered themselves good at performing MI.

**Table 2:** Reliability of MI subjective ability assessments and correlations between MI subjective ability and index of MI pre/post change in HDPV (*n* = 30)

	MIQ-RS	Reaching	Ease	Vividness	Frequency	General	Index of MI Pre/Post change
MIQ-RS	1	***r* = 0.71,** ***p* < 0.001**	**ρ = 0.50,** ***p* = 0.005**	*r* = 0.33, *p* = 0.073	ρ = 0.18, *p* = 0.350	***r* = 0.68,** ***p* < 0.001**	*r* = −0.20, *p* = 0.288
Reaching		1	ρ = 0.33, *p* = 0.072	*r* = 0.27, *p* = 0.152	ρ = −0.01, *p* = 0.967	***r* = 0.58,** ***p* < 0.001**	*r* = −0.15, *p* = 0.417
Ease			1	**ρ = 0.76,** ***p* < 0.001**	**ρ = 0.56,** ***p* = 0.001**	**ρ = 0.88,** ***p* < 0.001**	ρ = −0.18, *p* = 0.338
Vividness				1	**ρ = 0.63,** ***p* < 0.001**	***r* = 0.84,** ***p* < 0.001**	*r* = −0.03, *p* = 0.881
Frequency					1	**ρ = 0.66,** ***p* < 0.001**	ρ = −0.15, *p* = 0.424
General		** **	** **	** **		1	*r* = −0.22, *p* = 0.253
Index of MI Pre/Post change							1
ω if item dropped	0.77	0.8	0.7	0.7	0.75		

Correlation coefficients correspond to Pearson’s test for normally distributed variables and to Spearman’s test if normality was violated (significant Shapiro–Wilk’s test). Significant correlations are reported in bold.

A bivariate correlation analysis revealed no significant correlation between the general MI ability score and the Index of Pre/Post change in HDPV in the MI condition (*r* = −0.22, *p* = 0.253; Table 2). Thus, the subjective ability to perform MI did not strongly predict the magnitude of the PRB in the MI condition.

As a more exploratory analysis, the propensity for participants to mistakenly move during MI trials (although infrequent) could be considered a potential index of “excitability” on MI trials, which may have mediated the PRBs. To test that, the mean proportion of rejected MI trials was correlated with the Pre/Post change in HDPV in the MI condition. Separately, it was also correlated with the general MI ability score. Both correlations turned out to be nonsignificant (ρ = 0.09, *p* = 0.650; and ρ = 0.19, *p* = 0.321, respectively).

### Potential influence of conditions on other kinematic data

The next analysis sought to evaluate the influence of the Intermediate trial, which varied across conditions, on the subsequent movement. Namely, we compared RT and MT on Post trials relative to the Pre trials. RM-ANOVAs with factors Condition, Rotation, and Target were conducted on Pre/Post changes in RT and MT.

For RT, the RM-ANOVA only revealed a main effect of Condition (*F*_(2,58)_ = 6.01, *p* = 0.004, η^2^_p_ = 0.17) and Target (*F*_(2,58)_ = 5.59, *p* = 0.006, η^2^_p_ = 0.16), all other interactions and main effects being nonsignificant (all *p*-values > 0.073). When pooled by Conditions, data revealed negative Pre/Post changes in RT in all conditions, indicating that participants reacted faster from Pre to Post trials regardless of the condition (EXE: −15 ± 15 ms; MI: −9 ± 19 ms; CTL: −5 ± 16 ms). Bonferroni-corrected pairwise comparisons of the factor Condition revealed that the Pre/Post change in RT was significantly different between EXE and CTL (*t*_(29)_ = 3.29, *p*_Bonferroni_ = 0.008, *d*_z_ = 0.60). However, there was no difference between EXE and MI (*t*_(29)_ = 1.87, *p*_Bonferroni_ = 0.216, *d*_z_ = 0.34), nor between CTL and MI (*t*_(29)_ = 1.67, *p*_Bonferroni_ = 0.316, *d*_z_ = 0.30). Thus, participants tended to react faster on the third trial of a triplet than on the first one in all conditions, but significantly more so in the EXE condition compared with the CTL condition. This may be because the context (i.e., task instructions) did not change in the EXE triplets between the Pre to the Post trials, whereas it did in the MI and CTL triplets.

For MT, a significant Condition × Rotation interaction was found (*F*_(4,116)_ = 2.74, *p* = 0.032, η^2^_p_ = 0.09) as well as main effects of Condition (*F*_(2,58)_ = 3.76, *p* = 0.029, η^2^_p_ = 0.11) and Target (*F*_(2,58)_ = 3.97, *p* = 0.024, η^2^_p_ = 0.12), all other interactions and main effects being nonsignificant (all *p*-values > 0.080). Breakdown of the Condition × Rotation interaction revealed no simple effect of Rotation for any of the three Conditions, although there was a trend for MI (EXE: *F*_(2,58)_ = 2.02, *p*_HF_ = 0.148, η^2^_p_ = 0.07; MI: *F*_(2,58)_ = 3.05, *p* = 0.055, η^2^_p_ = 0.10; CTL: *F*_(2,58)_ = 1.22, *p* = 0.304, η^2^_p_ = 0.04), suggesting that Pre/Post changes in MT were similar between Rotations across Conditions and cannot account for the Pre/Post changes in HDPV. When split by Condition, data revealed no Pre/Post change in MT for EXE (0 ± 2 ms) and slight positive Pre/Post changes for MI (+2 ± 3 ms) as well as CTL (+1 ± 4 ms). Bonferroni-corrected pairwise comparisons of the Condition factor revealed that Pre/Post changes in MT were significantly different between EXE and MI (*t*_(29)_ = 2.66, *p*_Bonferroni_ = 0.038, *d*_z_ = 0.49). However, there was no difference between EXE and CTL (*t*_(29)_ = 2.04, *p*_Bonferroni_ = 0.152, *d*_z_ = 0.37), nor between MI and CTL (*t*_(29)_ = 0.43, *p*_Bonferroni_ = 1, *d*_z_ = 0.08). Thus, participants tended to move slower on the third trial of a triplet than on the first one in the MI and CTL conditions, and significantly more so in the MI condition than the EXE condition. Again, this may be because of the fact that the context did not change in the EXE triplets, whereas it did in the MI and CTL triplets.

### Muscular activity during intermediate trials

**Figure 3.**
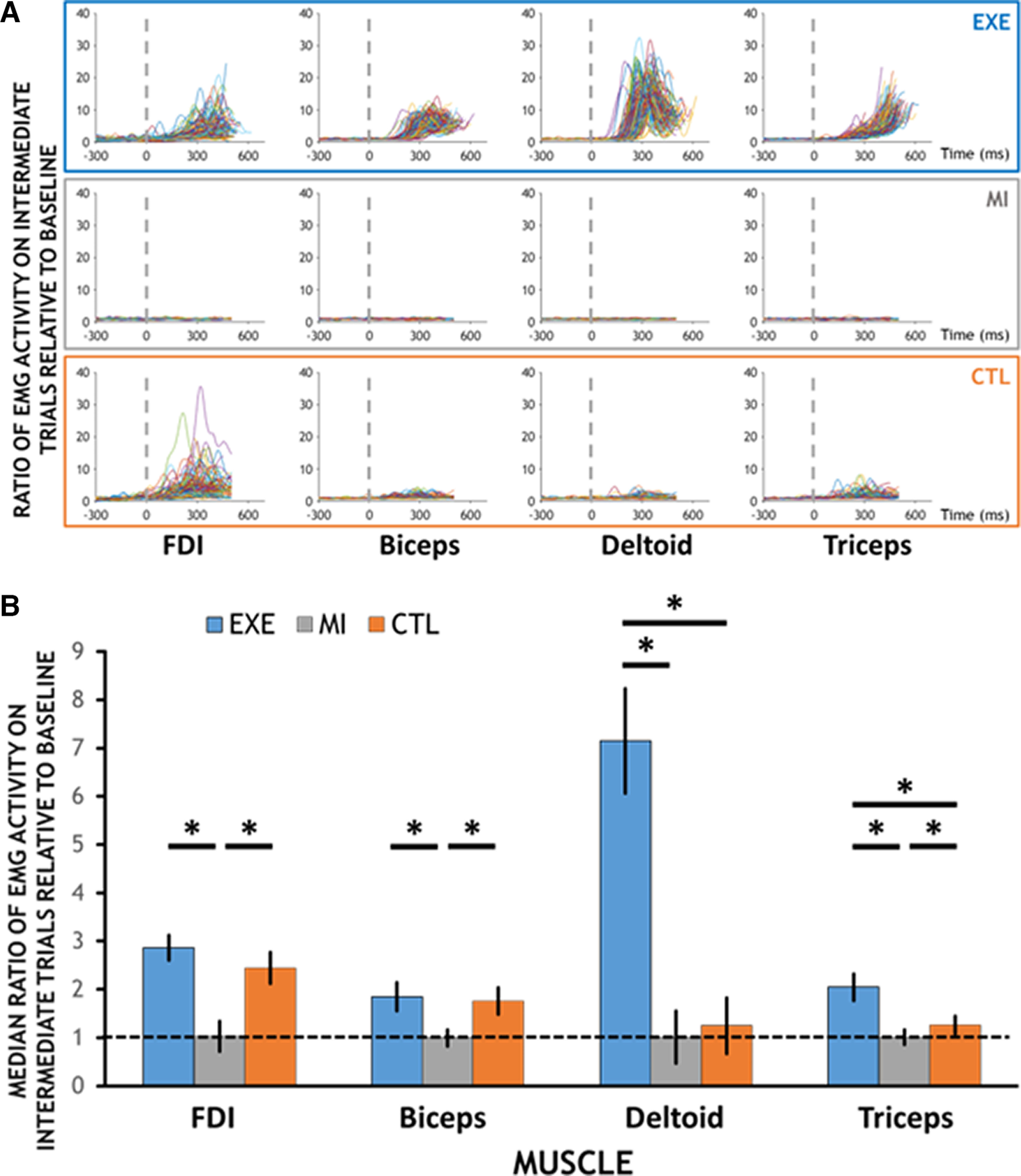
Assessment of EMG activity on intermediate trials. ***A***, EMG activity during Intermediate trials from one representative participant, for each Muscle (column) and Condition (row). The dashed line represents the time of target onset. ***B***, Median ratio of EMG activity on Intermediate trials relative to baseline, averaged across participants, for each muscle and condition. The dashed line represents a value of 1, which corresponds to EMG activity during the premovement baseline period. Error bars correspond to a 95% confidence interval. Significant differences are reported (*).

Figure 3*A* presents the EMG activity during Intermediate trials from a representative participant, for each Muscle and Condition. As can be seen, the EXE condition was associated with potent EMG activity in all four muscles, the CTL condition was associated with activity primarily in FDI and biceps, and most importantly, MI did not elicit discernable EMG activity, or at least no different from baseline in all four muscles.

We then statistically compared the ratio of EMG activity of Intermediate trials relative to baseline (300 ms before target onset) between conditions (Fig. 3*B*). This was done with a series of Wilcoxon signed-rank tests, since EMG ratios were not normally distributed. Results revealed that for the FDI, EMG was significantly greater in EXE (2.9 ± 1.4) than MI (1.0 ± 0.2), and in CTL (2.4 ± 1.6) than MI, but not in EXE than CTL (EXE vs MI: *W* = 0.00, *p*_Bonferroni_ < 0.001, RBC = 1.00; CTL vs MI: *W* = 3.00, *p*_Bonferroni_ < 0.001, RBC = 0.99; EXE vs CTL: *W* = −309.00, *p*_Bonferroni_ = 0.357, RBC = 0.33). Similarly, in the biceps, EMG was significantly greater in EXE (1.9 ± 1.1) than MI (1.0 ± 0.03), in CTL (1.8 ± 1.0) than MI, but not in EXE than CTL (EXE vs MI: *W* = 6.00, *p*_Bonferroni_ < 0.001, RBC = 0.97; CTL vs MI: *W* = 1.00, *p*_Bonferroni_ < 0.001, RBC = 1.00; EXE vs CTL: *W* = −267.00, *p*_Bonferroni_ = 1, RBC = 0.15). As for the deltoid, EMG was significantly greater in EXE (7.2 ± 4.5) than MI (1.0 ± 0.04), in EXE than CTL (1.3 ± 0.9), while CTL and MI did not differ from each other (EXE vs MI: *W* = 0.00, *p*_Bonferroni_ < 0.001, RBC = 1.00; CTL vs MI: *W* = 194.00, *p*_Bonferroni_ = 1.000, RBC = 0.17; EXE vs CTL: *W* = −465.00, *p*_Bonferroni_ < 0.001, RBC = 1.00;). Finally, for the triceps, EMG was found significantly greater in EXE (2.0 ± 1.1) than MI (1.0 ± 0.04), CTL (1.3 ± 0.5) than MI, and also in EXE than CTL (EXE vs MI: *W* = 0.00, *p*_Bonferroni_ < 0.001, RBC = 1.00; CTL vs MI: *W* = 31.00, *p*_Bonferroni_ < 0.001, RBC = 0.87; EXE vs CTL: *W* = 407.00, *p*_Bonferroni_ < 0.001, RBC = 0.75). These results show that, in addition to not physically moving, participants elicited minimal muscular activity in MI trials.

To statistically confirm that EMG activity on MI trials was indeed absent, we compared the ratio of EMG activity on MI trials against 1 (i.e., baseline). A value of 1 means that EMG activity was similar during baseline and MI periods. Wilcoxon signed-rank tests were again used and revealed that for each of the FDI, biceps, deltoid, and triceps muscles, muscular activity on MI trials was not different from 1 (respectively: *W* = 227.00, *p* = 0.919, RBC = −0.02; *W* = 182.00, *p* = 0.308, RBC = −0.22; *W* = 253.00, *p* = 0.685, RBC = 0.09; *W* = 182.00, *p* = 0.308, RBC = −0.22). These results show that participants did not elicit more muscular activity during MI trials than during the premovement baseline period.

## Discussion

The objective of this work was to determine whether MI is sufficient to induce implicit sensorimotor adaptation. Directional biases were assessed following intermittent exposure to ±30° visuomotor rotations, when participants either executed a center-out reaching movement (EXE), merely imagined it (MI), or performed a stereotyped inward movement to prevent MI (CTL). Interestingly, results revealed the presence of PRBs in all conditions. Nevertheless, the largest PRBs were found in the EXE condition, while MI and CTL conditions led to similarly smaller PRBs ∼1/3 in size. These results suggest that implicit sensorimotor adaptation can be partially supported by processes other than those that strictly pertain to generating motor commands. However, movement execution does potentiate it, pointing to additional contributions from motor commands and/or dynamic proprioceptive signals.

### Implicit adaptation occurs in absence of specific movement execution

The main finding of the present work is that adaptation occurred in absence of movement execution. Indeed, in all conditions, reaching movements were biased from the first to the third trial of perturbed triplets, in a direction opposite to the perturbation experienced on the Intermediate trial. This is likely to reflect implicit adaptation, as the magnitude of the direction-dependent PRBs in the current EXE condition was ∼7% of the induced rotation, a degree of directional change that is consistent with previous work quantifying the rate of early implicit visuomotor adaptation (McDougle et al., 2015; Morehead et al., 2017; H.E. Kim et al., 2018; Savoie et al., 2020). Given that we did not ask participants to report where they were aiming before each trial (Taylor et al., 2014), we cannot confirm that no strategy was used. Still, the current instructions to ignore the cursor and directly aim to the target have been shown to prevent explicit strategic mechanisms and to specifically engage implicit adaptation (Morehead et al., 2017; Savoie et al., 2020; Maresch et al., 2021; O.A. Kim et al., 2022). It is also known that other model-free processes such as use-dependent plasticity (directional bias toward the repeated movement) and operant reinforcement (association of the adapted movement with successful error reduction) can influence motor behavior in adaptation contexts (Huang et al., 2011). However, use-dependent plasticity would not be expected to contribute to a change in reach direction following a perturbed trial, but rather act against a PRB. Similarly, any influence of operant reinforcement is likely to be minimal in a context in which there are no systematic performance improvements across trials. It can thus be conjectured that the procedures conducted herein induced implicit adaptation, and that PRBs were a genuine reflection of it.

As reasoned in the introduction, significant PRBs were hypothesized to occur in the MI condition because of preparatory processes associated with MI (Kilteni et al., 2018). This result would lead to the interpretation that merely imagining oneself move to a target generates sensory predictions, which are then compared with the animated visual feedback and are sufficient to generate a SPE, ultimately leading to the observed PRBs. This result is qualitatively similar to that of a recent study (O.A. Kim et al., 2022) in which the authors also tested the necessity of movement execution for inducing implicit visuomotor adaptation. Using a similar protocol, the authors assessed PRBs in a condition in which participants executed the reaching movement compared with one in which participants planned the movement but were given a cue to withhold it just before execution (“no-go” trials). Results were strikingly similar to the present ones, as the no-movement condition also led to significant PRBs. Furthermore, their magnitude was ∼1/3 of those in the execution condition, a relative difference that is highly comparable to the one observed here. In adequation to the above interpretation, the authors argued that sensory predictions can be generated merely from a movement intent (or plan or goal) and lead to SPEs.

The preceding interpretation is anchored in the notion that preparatory processes linked to MI (or to planning and withholding as in O.A. Kim et al., 2022) contribute to the PRBs. However, this view is harder to reconcile with the fact that PRBs were also observed in the CTL condition. Indeed, in this condition, participants were explicitly told not to perform MI, and rather to make a stereotyped inward movement following target appearance, regardless of its location. The intuition behind this condition was to actively prevent participants from performing MI by “monopolizing” their motor system through the execution of a movement in a direction unrelated to that of the target. This was confirmed by the inward movement that was physically recorded on all trials that were kept for analysis, as well as the pattern of EMG activity that was qualitatively different from both the EXE and MI conditions. Hence, while it is impossible to confirm that participants were not performing imagery in the CTL condition, it is clear that their motor system was in a different state since the motor output differed. The fact that PRBs of similar magnitude were observed in the MI and CTL conditions thus does not support a specific role of MI per se, and rather forces us to consider other mechanistic explanations that would be common to both of them.

One possibility is that sensory predictions were generated as a result of the target appearance, which may have automatically engaged planning-related activity in the motor system, akin to an affordance (Cisek, 2007; Pezzulo and Cisek, 2016). The notion of affordance proposes that target objects are directly encoded into action opportunities within the motor network. In support, neuroimaging studies during reaching tasks have shown that target appearance elicits specific activity in the dorsal fronto-parietal cortex, even in a context in which one does not intend to move (Bernier et al., 2012, 2017). In this light, the target appearance may have engaged neural circuitry related to planning a movement toward it, and by extension perhaps also its predicted sensory outcomes, even in the CTL condition. In a sense, it may be that in the CTL condition, participants obligatorily planned a movement to the target, then had to inhibit that plan before executing the irrelevant reach. Under that possibility, the CTL condition may be considered similar to the no-go trials used in O.A. Kim et al. (2022), which may explain the similar pattern of results. We further note that this effect is likely to have been particularly potent in the present study as the displayed targets were the goal of a reaching movement on a very large majority of trials (∼89%).

Another possibility relates to the fact that participants were instructed to perform an eye movement toward the appearing target on all trials and all conditions. It may be that information related to the motor command to the extra-ocular muscles was transmitted to the limb motor system to jointly plan a hand movement, along with potential predictive signals. An interaction between motor commands sent to the eye and the hand is supported by neurophysiology, as there is overlap between saccade and reach fields in the posterior parietal cortex (Levy et al., 2007). Functionally, it is also known that foveating a target during reaching helps to guide the hand to it and to detect movement errors, enhancing the accuracy of the reach (de Brouwer et al., 2021). Hence, the present results may be because of the fact that bringing the eye to the target may have also engaged the arm reaching system, producing SPEs even in the CTL condition.

The above-mentioned possibilities are based on the idea that the MI and CTL conditions are associated with sensory predictions, linked to either the target appearance or the ocular motor commands, which lead to SPEs and ultimately PRBs. Another distinct possibility is that the PRBs in these conditions were related to something else than a SPE. One candidate is the target error (i.e., the mismatch between the end-position of the cursor and the target) which, by design, was common to all conditions given the imposed visuomotor rotations. It could be that merely seeing the cursor deviate away form the target generates a form of violation from expectancy, which leads to an automatic correction on the subsequent movement. This may be a purely sensory or attentional phenomenon, independent from whether a movement was actually produced on the perturbed trial. This would find echo in literature showing that implicit adaptation is driven not solely by the SPE, but also by task outcome. Namely, recent studies have manipulated target size to create contexts in which an SPE is paired with either a target hit or a target miss (H.E. Kim et al., 2019; Tsay et al., 2022). They have revealed that the magnitude of behavioral change is reduced on target hits, suggesting that implicit adaptation is the result of these two interacting error signals. In such a scheme, the partial PRBs in the MI and CTL conditions may reflect the exclusive contribution of a learning process that is driven by target errors, while the more potent PRBs in the EXE condition would reflect the summed effect of the SPEs and the target errors.

### Potentiation of adaptation by movement execution

The present work also shows that PRBs were larger in the EXE condition compared with the MI or CTL conditions. This indicates that implicit adaptation benefits from execution-related factors. A straighforward explanation is that descending motor commands are necessary to generate sensory predictions. While this view generally agrees with most current theoretical models (Wolpert et al., 1998; Wolpert and Ghahramani, 2000; Krakauer et al., 2019), it nonetheless stands in contrast with recent literature showing that adaptation depends more on preparatory movement dynamics than those related to execution (Sheahan et al., 2016). It is also inconsistent with the finding that MI generates sensory predictions of sufficient fidelity to lead to sensory attenuation in a force-matching task (Kilteni et al., 2018).

One way to reconcile these lines of evidence is that sensory predictions may indeed emanate from processes linked to movement preparation more so than execution, but be made more precise when a movement is planned and executed compared with simply planned (i.e., imagined). Indeed, planning and executing a stream of descending motor commands may allow to more precisely anticipate the upcoming sensory inflow, both spatially and temporally, ultimately affording a tighter coupling between the sensory predictions and their associated sensory consequences. In opposition, it is likely that although participants perceived themselves as good performers of MI in the present study, there must have been a certain degree of variability in the spatiotemporal accuracy and vividness of the movement sensations evoked through MI. In light of the finding that implicit adaptation is reduced on introduction of a temporal delay between an action and its visual feedback (Kitazawa et al., 1995; Honda et al., 2012), it would follow that the more spatiotemporally precise the predictions, the more salient the SPEs, and the more potent the PRBs. Mechanistically, this could take the form of movement execution acting as a discrete event serving to synchronize neural activity in sensory regions of the brain. Evidence for this comes from the finding that low-frequency oscillations in visual regions are phase-reset by movement onset (Tomassini et al., 2017). Phase information even predicts perceptual performance up to 1 s before movement, indicating that movement initiation and perception are automatically synchronized since the early stages of motor planning.

Another possibility is that movement execution improves implicit adaptation through the presence of dynamic proprioceptive signals, which were only present in the EXE trials. It may be that the cross-sensory error signal (i.e., the discrepancy between visual and proprioceptive estimates of hand position) triggered a shift in the perceived sense of hand position (proprioceptive realignment), which also contributed to PRBs in the EXE trials (Körding and Wolpert, 2004; Salomonczyk et al., 2013; Maksimovic et al., 2020). There has recently been a greater appreciation of the contribution of proprioceptive cues to adaptation, namely, the degree to which proprioceptive realignment may contribute to implicit adaptive changes. For instance, mere exposure to a visuo-proprioceptive shift, even in a context in which the limb is passively moved by a robot away from a target, without any visual task error, is enough to trigger adaptive motor responses in subsequent voluntary reaches (Cressman and Henriques, 2010; Sakamoto and Kondo, 2015). In this context, the change in felt hand position was correlated with the observed aftereffects, suggesting a potential common mechanism. In this light, it may be that the PRBs in the EXE condition reflected the joint contribution of SPE signals as well as that of the cross-sensory error signal, whereas the latter signal is likely to have been much weaker in the other conditions given the absence of movement.

While more work is needed to uncover the exact neurophysiological mechanisms at play, the present work adds to the growing body of evidence suggesting that implicit adaptation may not strictly necessitate overt execution, but be supported to some degree by movement preparation alone (Sheahan et al., 2016, 2018; O.A. Kim et al., 2022). This opens up the interesting possibility that in certain pathologic conditions (such as hypometria), pairing an appropriate stimulus with carefully-manipulated visual feedback (such as a gain), may be used as a tool to alter movements in a behaviorally relevant way.

## Synthesis

Reviewing Editor: David Franklin, Technische Universitat Munchen Decisions are customarily a result of the Reviewing Editor and the peer reviewers coming together and discussing their recommendations until a consensus is reached. When revisions are invited, a fact-based synthesis statement explaining their decision and outlining what is needed to prepare a revision will be listed below. The following reviewer(s) agreed to reveal their identity: Raphael Schween.

Reviewer 1:

The authors have done a nice job of responding to my comments as well as the other reviewer’s key points about data analysis.

Reviewer 2:

I thank the authors for addressing all of my previous concerns, which I find they have done in an overall convincing manner. Whereas the authors understandably hold that adding extensive controls to understand the processes underlying adaptation in the control group (and by extension whether there is anything specific to the Mi group) is beyond the focus of the current work, I find that the changes to the presentation of MI versus CTL now better reflect these remaining knowledge gaps. Furthermore, the addition of trajectories, the analysis of movement endpoint, and the use of sign-inverted instead of absolute value index have strengthened my belief in the data. I also find the authors’ consideration of other processes beyond adaptation of internal models based on sensory prediction errors convincing, and particularly so with regard to use-dependent and reinforcement components. I have no additional comments.
